# Aqua­bis[1-ethyl-6-fluoro-7-(4-methyl­piperazin-1-yl)-4-oxo-1,4-dihydro­quinoline-3-carboxyl­ato]zinc(II) dihydrate

**DOI:** 10.1107/S1600536807068298

**Published:** 2008-01-09

**Authors:** Wei Qi, Jing Huang, Zhe An

**Affiliations:** a School of Pharmaceutical Science, Harbin Medical University, Harbin 150086, People’s Republic of China

## Abstract

The title compound, [Zn(C_17_H_19_FN_3_O_3_)_2_(H_2_O)]·2H_2_O or [Zn(pef)_2_(H_2_O)]·2H_2_O, where pef is 1-ethyl-6-fluoro-7-(4-methyl­piperazin-4-yl)-4-oxo-1,4-dihydro­quinoline-3-carb­oxyl­ate, was synthesized under hydro­thermal conditions. The Zn^II^ atom exhibits a distorted ZnO_5_ square-pyramidal geometry defined by two bidentate *O*,*O*-bonded pef anions in the basal plane and one water mol­ecule in the apical position. A network of O—H⋯O and O—H⋯N hydrogen bonds is formed between the Zn^II^ complexes and the uncoordinated water mol­ecules.

## Related literature

For Ag^I^, Mn^II^ and Co^II^ complexes of the pef anion, see: Baenziger *et al.* (1986 ▶); An, Huang & Qi (2007 ▶); An, Qi & Huang (2007 ▶). For background on the medicinal uses of Hpef, see: Mizuki *et al.* (1996 ▶).
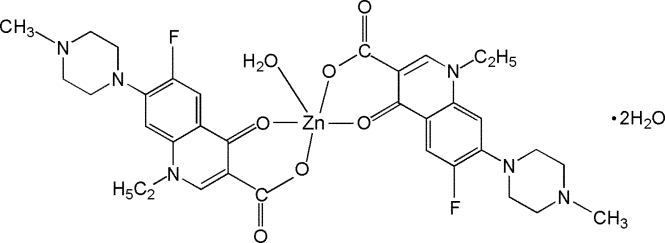


## Experimental

### 

#### Crystal data

[Zn(C_17_H_19_FN_3_O_3_)_2_(H_2_O)]·2H_2_O
*M* *_r_* = 784.12
Triclinic, 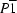

*a* = 10.0046 (6) Å
*b* = 10.9372 (6) Å
*c* = 18.2738 (14) Å
α = 96.933 (2)°
β = 102.839 (1)°
γ = 111.699 (1)°
*V* = 1765.4 (2) Å^3^
*Z* = 2
Mo *K*α radiation
μ = 0.77 mm^−1^
*T* = 293 (2) K
0.43 × 0.22 × 0.18 mm

#### Data collection

Bruker SMART CCD diffractometer
Absorption correction: multi-scan (*SADABS*; Sheldrick, 1996 ▶) *T*_min_ = 0.733, *T*_max_ = 0.874
9009 measured reflections
6138 independent reflections
4177 reflections with *I* > 2σ(*I*)
*R*_int_ = 0.029

#### Refinement

*R*[*F* ^2^ > 2σ(*F* ^2^)] = 0.056
*wR*(*F* ^2^) = 0.160
*S* = 1.01
6138 reflections
481 parameters
15 restraints
H atoms treated by a mixture of independent and constrained refinement
Δρ_max_ = 0.92 e Å^−3^
Δρ_min_ = −0.57 e Å^−3^

### 

Data collection: *SMART* (Bruker, 1998 ▶); cell refinement: *SAINT* (Bruker, 1998 ▶); data reduction: *SAINT*; program(s) used to solve structure: *SHELXS97* (Sheldrick, 2008 ▶); program(s) used to refine structure: *SHELXL97* (Sheldrick, 2008 ▶); molecular graphics: *SHELXTL* (Bruker, 1998 ▶); software used to prepare material for publication: *SHELXTL*.

## Supplementary Material

Crystal structure: contains datablocks I, global. DOI: 10.1107/S1600536807068298/bi2272sup1.cif

Structure factors: contains datablocks I. DOI: 10.1107/S1600536807068298/bi2272Isup2.hkl

Additional supplementary materials:  crystallographic information; 3D view; checkCIF report

## Figures and Tables

**Table 1:** Hydrogen-bond geometry (Å, °)

*D*—H⋯*A*	*D*—H	H⋯*A*	*D*⋯*A*	*D*—H⋯*A*
O7—H7⋯O5^i^	0.85 (1)	1.79 (1)	2.638 (5)	176 (6)
O8—H8*WA*⋯N5	0.85	2.21	3.063 (9)	179
O8—H8*WB*⋯O1^ii^	0.85	2.20	3.054 (7)	179
O9—H9*WA*⋯N6	0.85	1.99	2.843 (9)	180

Symmetry codes: (i) 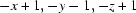; (ii) 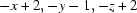.

**Fig. 1.**
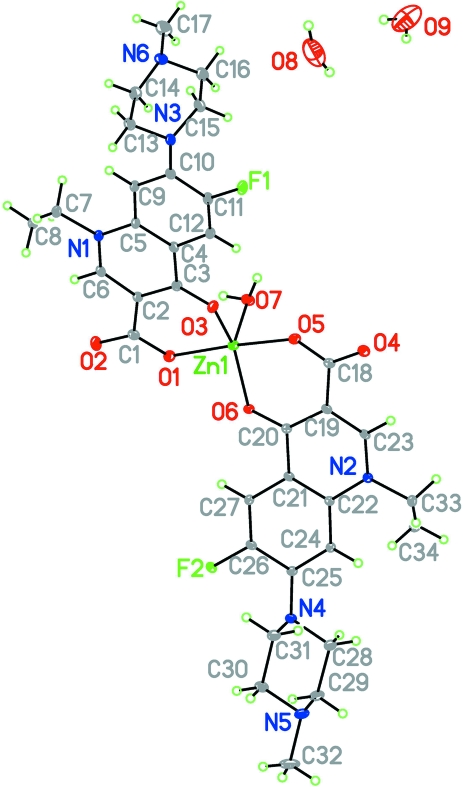
The asymmetric unit of the title compound showing the showing 50% displacement ellipsoids for non-H atoms.

## supplementary crystallographic information

### Comment

Pefloxacin (Hpef, C_17_H_20_FN_3_O_3_, 1-ethyl-6-fluoro-7-(4-methylpiperazin-1-yl)-4-oxo-quinoline-3-carboxylic acid) is member of a class of quinolones used to treat infections (Mizuki *et al.*, 1996). The silver(I), manganese(II) and cobalt(II) complexes of the pefloxacin (pef) anion have been reported (Baenziger *et al.*, 1986; An, Huang & Qi, 2007; An, Qi & Huang, 2007). The title zinc(II) complex is reported here.

The title structure of is built up from Zn^2+^ cations, pef ligands, one coordinated water molecule, and two uncoordinated water molecules (Fig. 1). The coordination geometry around Zn^II^ is a slightly distorted square pyramid (Table 1). The components of the structure are linked by O—H···O and O—H···N hydrogen bonds involving all the potential donors (Table 2).

### Experimental

A mixture of Zn(CH_3_COO)_2_.2H_2_O (0.055 g, 0.25 mmol), Hpef (0.17 g, 0.5 mmol) and water (12 ml) was stirred for 30 min in air. The mixture was then transferred to a 23 ml Teflon-lined hydrothermal bomb. The bomb was kept at 433 K for 72 h under autogenous pressure. Upon cooling, colorless prisms of the title compound were obtained from the reaction mixture.

### Refinement

All H atoms on C atoms were generated geometrically and refined as riding atoms with C—H = 0.93 Å and *U*_iso_(H) = 1.2*U*_eq_(C). H atoms of the coordinated water molecule were located from difference Fourier maps and refined with distance restraints of d(O—H) = 0.82 (1) Å and d(H···H) = 1.35 Å. H atoms of the lattice water molecules were placed so as to form a reasonable H-bond network and were refined as riding. Their positions are uncertain.

### Crystal data

**Table:**

[Zn(C_17_H_19_FN_3_O_3_)_2_(H_2_O)]·2H_2_O	*Z* = 2
*M**_r_* = 784.12	*F*_000_ = 820
Triclinic, *P*1	*D*_x_ = 1.475 Mg m^−^^3^
Hall symbol: -P 1	Mo *K*α radiation λ = 0.71073 Å
*a* = 10.0046 (6) Å	Cell parameters from 1700 reflections
*b* = 10.9372 (6) Å	θ = 2.4–22.1º
*c* = 18.2738 (14) Å	µ = 0.77 mm^−^^1^
α = 96.933 (2)º	*T* = 293 (2) K
β = 102.839 (1)º	Block, colorless
γ = 111.699 (1)º	0.43 × 0.22 × 0.18 mm
*V* = 1765.4 (2) Å^3^	

### Data collection

**Table:**

Bruker SMART CCD diffractometer	6138 independent reflections
Radiation source: fine-focus sealed tube	4177 reflections with *I* > 2σ(*I*)
Monochromator: graphite	*R*_int_ = 0.029
*T* = 293(2) K	θ_max_ = 25.0º
ω scans	θ_min_ = 2.3º
Absorption correction: multi-scan(SADABS; Sheldrick, 1996)	*h* = −11→11
*T*_min_ = 0.733, *T*_max_ = 0.874	*k* = −11→12
9009 measured reflections	*l* = −21→17

### Refinement

**Table:**

Refinement on *F*^2^	Secondary atom site location: difference Fourier map
Least-squares matrix: full	Hydrogen site location: inferred from neighbouring sites
*R*[*F*^2^ > 2σ(*F*^2^)] = 0.056	H atoms treated by a mixture of independent and constrained refinement
*wR*(*F*^2^) = 0.160	*w* = 1/[σ^2^(*F*_o_^2^) + (0.0881*P*)^2^] where *P* = (*F*_o_^2^ + 2*F*_c_^2^)/3
*S* = 1.01	(Δ/σ)_max_ < 0.001
6138 reflections	Δρ_max_ = 0.92 e Å^−^^3^
481 parameters	Δρ_min_ = −0.57 e Å^−^^3^
15 restraints	Extinction correction: none
Primary atom site location: structure-invariant direct methods	

### Special details

**Table:**

Geometry. All e.s.d.'s (except the e.s.d. in the dihedral angle between two l.s. planes) are estimated using the full covariance matrix. The cell e.s.d.'s are taken into account individually in the estimation of e.s.d.'s in distances, angles and torsion angles; correlations between e.s.d.'s in cell parameters are only used when they are defined by crystal symmetry. An approximate (isotropic) treatment of cell e.s.d.'s is used for estimating e.s.d.'s involving l.s. planes.

### Fractional atomic coordinates and isotropic or equivalent isotropic displacement parameters (Å^2^)

**Table:**

	*x*	*y*	*z*	*U*_iso_*/*U*_eq_	
Zn1	0.65571 (6)	−0.35549 (5)	0.62580 (3)	0.0396 (2)	
C1	0.9050 (6)	−0.0856 (5)	0.6456 (3)	0.0441 (11)	
C2	0.8074 (5)	−0.0651 (4)	0.5766 (2)	0.0353 (10)	
C3	0.6514 (5)	−0.1506 (4)	0.5394 (2)	0.0356 (10)	
C4	0.5825 (5)	−0.1181 (4)	0.4702 (2)	0.0347 (10)	
C5	0.6621 (5)	−0.0074 (4)	0.4427 (2)	0.0329 (10)	
C6	0.8756 (5)	0.0422 (4)	0.5464 (2)	0.0404 (11)	
H6A	0.9753	0.0988	0.5723	0.048*	
C7	0.9016 (5)	0.1918 (4)	0.4573 (3)	0.0436 (11)	
H7A	0.8818	0.1685	0.4019	0.052*	
H7B	1.0074	0.2151	0.4807	0.052*	
C8	0.8684 (7)	0.3127 (5)	0.4782 (3)	0.0605 (15)	
H8A	0.9279	0.3861	0.4594	0.091*	
H8B	0.8923	0.3389	0.5332	0.091*	
H8C	0.7637	0.2903	0.4554	0.091*	
C9	0.5920 (5)	0.0196 (4)	0.3743 (3)	0.0409 (11)	
H9A	0.6462	0.0927	0.3562	0.049*	
C10	0.4435 (5)	−0.0614 (4)	0.3337 (2)	0.0387 (10)	
C11	0.3675 (5)	−0.1721 (4)	0.3636 (3)	0.0437 (11)	
C12	0.4334 (5)	−0.2002 (4)	0.4280 (3)	0.0382 (10)	
H12A	0.3792	−0.2752	0.4447	0.046*	
C13	0.4351 (6)	0.0964 (5)	0.2524 (3)	0.0545 (13)	
H13A	0.5273	0.1077	0.2394	0.065*	
H13B	0.4599	0.1645	0.2983	0.065*	
C14	0.3267 (7)	0.1145 (5)	0.1864 (3)	0.0627 (15)	
H14A	0.2368	0.1078	0.2007	0.075*	
H14B	0.3729	0.2036	0.1767	0.075*	
C15	0.3138 (6)	−0.1427 (5)	0.1982 (3)	0.0574 (14)	
H15A	0.2586	−0.2284	0.2095	0.069*	
H15B	0.3991	−0.1481	0.1835	0.069*	
C16	0.2144 (7)	−0.1193 (6)	0.1324 (3)	0.0702 (17)	
H16A	0.1899	−0.1874	0.0865	0.084*	
H16B	0.1214	−0.1284	0.1437	0.084*	
C17	0.1944 (9)	0.0369 (7)	0.0515 (4)	0.103 (3)	
H17A	0.2496	0.1228	0.0416	0.154*	
H17B	0.1042	0.0361	0.0619	0.154*	
H17C	0.1687	−0.0334	0.0073	0.154*	
C18	0.3682 (5)	−0.5979 (5)	0.6171 (2)	0.0400 (11)	
C19	0.4394 (5)	−0.6025 (4)	0.6975 (2)	0.0331 (10)	
C20	0.5795 (5)	−0.5029 (4)	0.7459 (2)	0.0335 (10)	
C21	0.6207 (5)	−0.5154 (4)	0.8257 (2)	0.0324 (10)	
C22	0.5323 (5)	−0.6236 (4)	0.8515 (2)	0.0307 (9)	
C23	0.3610 (5)	−0.7083 (4)	0.7260 (2)	0.0351 (10)	
H23A	0.2727	−0.7754	0.6925	0.042*	
C24	0.5761 (5)	−0.6335 (4)	0.9281 (2)	0.0351 (10)	
H24A	0.5172	−0.7074	0.9439	0.042*	
C25	0.7051 (5)	−0.5354 (4)	0.9808 (2)	0.0333 (10)	
C26	0.7878 (5)	−0.4248 (4)	0.9528 (2)	0.0345 (10)	
C27	0.7521 (5)	−0.4143 (4)	0.8790 (2)	0.0346 (10)	
H27A	0.8132	−0.3413	0.8633	0.042*	
C28	0.6444 (5)	−0.6319 (5)	1.0889 (3)	0.0482 (12)	
H28A	0.6359	−0.7224	1.0711	0.058*	
H28B	0.5461	−0.6309	1.0710	0.058*	
C29	0.7005 (6)	−0.5917 (6)	1.1766 (3)	0.0615 (15)	
H29A	0.7050	−0.5024	1.1938	0.074*	
H29B	0.6299	−0.6545	1.1975	0.074*	
C30	0.9536 (5)	−0.4951 (5)	1.1756 (3)	0.0481 (12)	
H30A	1.0526	−0.4943	1.1942	0.058*	
H30B	0.9602	−0.4052	1.1933	0.058*	
C31	0.9024 (5)	−0.5326 (5)	1.0887 (3)	0.0460 (12)	
H31A	0.9727	−0.4670	1.0690	0.055*	
H31B	0.9013	−0.6203	1.0711	0.055*	
C32	0.8983 (8)	−0.5532 (9)	1.2909 (3)	0.103 (3)	
H32A	0.9915	−0.5615	1.3096	0.154*	
H32B	0.8235	−0.6120	1.3105	0.154*	
H32C	0.9123	−0.4616	1.3078	0.154*	
C33	0.3057 (5)	−0.8432 (4)	0.8210 (3)	0.0437 (12)	
H33A	0.3692	−0.8726	0.8562	0.052*	
H33B	0.2492	−0.9160	0.7756	0.052*	
C34	0.1988 (6)	−0.8150 (6)	0.8587 (3)	0.0674 (16)	
H34A	0.1430	−0.8936	0.8750	0.101*	
H34B	0.1306	−0.7926	0.8226	0.101*	
H34C	0.2540	−0.7407	0.9025	0.101*	
F1	0.2212 (3)	−0.2509 (3)	0.32586 (17)	0.0646 (9)	
F2	0.9109 (3)	−0.3219 (2)	1.00377 (13)	0.0482 (7)	
N1	0.8111 (4)	0.0726 (3)	0.4827 (2)	0.0377 (9)	
N2	0.4017 (4)	−0.7226 (3)	0.79826 (19)	0.0349 (8)	
N3	0.3679 (4)	−0.0365 (4)	0.2670 (2)	0.0454 (10)	
N4	0.7503 (4)	−0.5368 (3)	1.05850 (19)	0.0363 (9)	
N5	0.8486 (5)	−0.5911 (5)	1.2059 (2)	0.0573 (11)	
N6	0.2865 (5)	0.0144 (5)	0.1179 (2)	0.0587 (12)	
O1	0.8523 (4)	−0.1942 (3)	0.66928 (18)	0.0512 (9)	
O2	1.0321 (4)	0.0026 (4)	0.6755 (2)	0.0689 (11)	
O3	0.5724 (3)	−0.2508 (3)	0.56177 (18)	0.0441 (8)	
O4	0.2415 (4)	−0.6836 (3)	0.58155 (18)	0.0567 (9)	
O5	0.4425 (4)	−0.5024 (4)	0.58903 (18)	0.0594 (10)	
O6	0.6706 (4)	−0.4034 (3)	0.72757 (16)	0.0494 (9)	
O7	0.7300 (4)	−0.4547 (4)	0.55184 (19)	0.0488 (8)	
H7	0.672 (5)	−0.467 (6)	0.5075 (15)	0.08 (2)*	
H8	0.814 (3)	−0.394 (5)	0.553 (3)	0.10 (3)*	
O8	0.9310 (10)	−0.8323 (6)	1.1768 (4)	0.202 (4)	
H8WA	0.9083	−0.7651	1.1844	0.302*	
H8WB	0.9920	−0.8251	1.2193	0.302*	
O9	0.5346 (9)	−0.0015 (8)	0.0726 (5)	0.177 (3)	
H9WA	0.4606	0.0034	0.0863	0.266*	
H9WB	0.5699	−0.0042	0.0344	0.266*	

### Atomic displacement parameters (Å^2^)

**Table:**

	*U*^11^	*U*^22^	*U*^33^	*U*^12^	*U*^13^	*U*^23^
Zn1	0.0441 (3)	0.0411 (3)	0.0264 (3)	0.0110 (2)	0.0057 (2)	0.0112 (2)
C1	0.047 (3)	0.046 (3)	0.031 (3)	0.015 (2)	0.003 (2)	0.007 (2)
C2	0.035 (2)	0.033 (2)	0.029 (2)	0.0085 (19)	0.0021 (19)	0.0082 (18)
C3	0.035 (2)	0.035 (2)	0.037 (2)	0.015 (2)	0.010 (2)	0.009 (2)
C4	0.033 (2)	0.031 (2)	0.035 (2)	0.0106 (19)	0.006 (2)	0.0052 (18)
C5	0.029 (2)	0.026 (2)	0.038 (2)	0.0061 (18)	0.0050 (19)	0.0091 (18)
C6	0.033 (2)	0.037 (3)	0.034 (2)	0.003 (2)	−0.003 (2)	0.002 (2)
C7	0.031 (2)	0.043 (3)	0.044 (3)	0.003 (2)	0.005 (2)	0.014 (2)
C8	0.075 (4)	0.039 (3)	0.050 (3)	0.003 (3)	0.017 (3)	0.013 (2)
C9	0.042 (3)	0.031 (2)	0.041 (3)	0.007 (2)	0.007 (2)	0.012 (2)
C10	0.038 (3)	0.034 (2)	0.035 (2)	0.011 (2)	0.000 (2)	0.0052 (19)
C11	0.027 (2)	0.041 (3)	0.048 (3)	0.005 (2)	−0.001 (2)	0.010 (2)
C12	0.029 (2)	0.037 (2)	0.040 (3)	0.005 (2)	0.008 (2)	0.012 (2)
C13	0.062 (3)	0.045 (3)	0.045 (3)	0.018 (3)	0.000 (3)	0.011 (2)
C14	0.064 (4)	0.055 (3)	0.064 (4)	0.024 (3)	0.003 (3)	0.021 (3)
C15	0.057 (3)	0.047 (3)	0.051 (3)	0.013 (3)	0.000 (3)	0.007 (2)
C16	0.061 (4)	0.068 (4)	0.050 (3)	0.010 (3)	−0.012 (3)	0.008 (3)
C17	0.116 (6)	0.100 (5)	0.066 (4)	0.044 (5)	−0.026 (4)	0.020 (4)
C18	0.045 (3)	0.042 (3)	0.028 (2)	0.015 (2)	0.007 (2)	0.007 (2)
C19	0.031 (2)	0.036 (2)	0.025 (2)	0.0098 (19)	0.0027 (19)	0.0014 (18)
C20	0.037 (2)	0.032 (2)	0.028 (2)	0.011 (2)	0.010 (2)	0.0064 (18)
C21	0.031 (2)	0.036 (2)	0.023 (2)	0.0097 (19)	0.0040 (18)	0.0026 (18)
C22	0.031 (2)	0.029 (2)	0.025 (2)	0.0075 (18)	0.0042 (18)	0.0046 (17)
C23	0.032 (2)	0.038 (2)	0.023 (2)	0.0083 (19)	−0.0010 (19)	−0.0006 (18)
C24	0.037 (2)	0.031 (2)	0.029 (2)	0.0049 (19)	0.009 (2)	0.0075 (18)
C25	0.034 (2)	0.037 (2)	0.025 (2)	0.0109 (19)	0.0058 (19)	0.0095 (18)
C26	0.031 (2)	0.039 (2)	0.023 (2)	0.0079 (19)	0.0018 (19)	0.0027 (18)
C27	0.036 (2)	0.030 (2)	0.030 (2)	0.0043 (19)	0.009 (2)	0.0082 (18)
C28	0.041 (3)	0.058 (3)	0.037 (3)	0.011 (2)	0.007 (2)	0.020 (2)
C29	0.053 (3)	0.088 (4)	0.039 (3)	0.017 (3)	0.016 (3)	0.030 (3)
C30	0.041 (3)	0.064 (3)	0.032 (3)	0.020 (2)	0.000 (2)	0.009 (2)
C31	0.039 (3)	0.063 (3)	0.035 (3)	0.018 (2)	0.010 (2)	0.017 (2)
C32	0.087 (5)	0.172 (8)	0.034 (3)	0.036 (5)	0.010 (3)	0.041 (4)
C33	0.039 (3)	0.034 (2)	0.040 (3)	−0.001 (2)	0.002 (2)	0.009 (2)
C34	0.058 (4)	0.065 (4)	0.081 (4)	0.017 (3)	0.035 (3)	0.025 (3)
F1	0.0316 (15)	0.0632 (19)	0.0650 (19)	−0.0069 (14)	−0.0104 (14)	0.0259 (15)
F2	0.0408 (15)	0.0463 (15)	0.0287 (14)	−0.0043 (12)	−0.0032 (12)	0.0033 (11)
N1	0.030 (2)	0.036 (2)	0.033 (2)	0.0028 (16)	0.0018 (17)	0.0088 (16)
N2	0.033 (2)	0.0335 (19)	0.0283 (19)	0.0057 (16)	0.0042 (16)	0.0056 (15)
N3	0.047 (2)	0.035 (2)	0.037 (2)	0.0076 (18)	−0.0031 (19)	0.0106 (17)
N4	0.031 (2)	0.044 (2)	0.0250 (19)	0.0082 (17)	0.0037 (16)	0.0092 (16)
N5	0.057 (3)	0.076 (3)	0.036 (2)	0.021 (2)	0.008 (2)	0.029 (2)
N6	0.066 (3)	0.064 (3)	0.036 (2)	0.024 (2)	−0.001 (2)	0.012 (2)
O1	0.048 (2)	0.050 (2)	0.0382 (19)	0.0058 (16)	−0.0005 (16)	0.0188 (16)
O2	0.045 (2)	0.060 (2)	0.056 (2)	−0.0112 (18)	−0.0173 (18)	0.0176 (19)
O3	0.0338 (17)	0.0449 (18)	0.050 (2)	0.0094 (15)	0.0090 (15)	0.0234 (16)
O4	0.044 (2)	0.062 (2)	0.0362 (19)	0.0011 (18)	−0.0042 (16)	0.0091 (16)
O5	0.057 (2)	0.062 (2)	0.0329 (18)	0.0001 (18)	0.0008 (17)	0.0185 (17)
O6	0.050 (2)	0.0478 (19)	0.0239 (16)	−0.0036 (16)	0.0012 (15)	0.0107 (14)
O7	0.047 (2)	0.059 (2)	0.0323 (19)	0.0187 (19)	0.0018 (17)	0.0074 (16)
O8	0.278 (8)	0.100 (4)	0.153 (5)	0.064 (5)	−0.051 (5)	0.028 (4)
O9	0.205 (6)	0.206 (6)	0.208 (6)	0.133 (5)	0.109 (5)	0.106 (5)

### Geometric parameters (Å, °)

**Table:**

Zn1—O6	1.981 (3)	C18—O5	1.278 (5)
Zn1—O3	1.993 (3)	C18—C19	1.499 (6)
Zn1—O1	2.004 (3)	C19—C23	1.371 (6)
Zn1—O5	2.037 (3)	C19—C20	1.422 (6)
Zn1—O7	2.061 (4)	C20—O6	1.272 (5)
C1—O2	1.226 (5)	C20—C21	1.461 (6)
C1—O1	1.280 (5)	C21—C22	1.398 (6)
C1—C2	1.508 (6)	C21—C27	1.413 (6)
C2—C6	1.371 (6)	C22—N2	1.400 (5)
C2—C3	1.438 (6)	C22—C24	1.401 (6)
C3—O3	1.267 (5)	C23—N2	1.339 (5)
C3—C4	1.451 (6)	C23—H23A	0.930
C4—C12	1.399 (6)	C24—C25	1.385 (6)
C4—C5	1.401 (6)	C24—H24A	0.930
C5—N1	1.385 (5)	C25—N4	1.395 (5)
C5—C9	1.413 (6)	C25—C26	1.416 (6)
C6—N1	1.342 (5)	C26—C27	1.348 (6)
C6—H6A	0.930	C26—F2	1.365 (4)
C7—N1	1.481 (5)	C27—H27A	0.930
C7—C8	1.506 (7)	C28—N4	1.448 (5)
C7—H7A	0.970	C28—C29	1.525 (6)
C7—H7B	0.970	C28—H28A	0.970
C8—H8A	0.960	C28—H28B	0.970
C8—H8B	0.960	C29—N5	1.457 (7)
C8—H8C	0.960	C29—H29A	0.970
C9—C10	1.386 (6)	C29—H29B	0.970
C9—H9A	0.930	C30—N5	1.448 (6)
C10—N3	1.397 (5)	C30—C31	1.508 (6)
C10—C11	1.416 (6)	C30—H30A	0.970
C11—C12	1.345 (6)	C30—H30B	0.970
C11—F1	1.354 (5)	C31—N4	1.481 (6)
C12—H12A	0.930	C31—H31A	0.970
C13—N3	1.446 (6)	C31—H31B	0.970
C13—C14	1.518 (7)	C32—N5	1.477 (7)
C13—H13A	0.970	C32—H32A	0.960
C13—H13B	0.970	C32—H32B	0.960
C14—N6	1.431 (6)	C32—H32C	0.960
C14—H14A	0.970	C33—N2	1.486 (5)
C14—H14B	0.970	C33—C34	1.494 (7)
C15—N3	1.451 (6)	C33—H33A	0.970
C15—C16	1.494 (7)	C33—H33B	0.970
C15—H15A	0.970	C34—H34A	0.960
C15—H15B	0.970	C34—H34B	0.960
C16—N6	1.459 (7)	C34—H34C	0.960
C16—H16A	0.970	O7—H7	0.85 (1)
C16—H16B	0.970	O7—H8	0.85 (1)
C17—N6	1.458 (7)	O8—H8WA	0.85
C17—H17A	0.960	O8—H8WB	0.85
C17—H17B	0.960	O9—H9WA	0.85
C17—H17C	0.960	O9—H9WB	0.85
C18—O4	1.231 (5)		
			
O6—Zn1—O3	141.09 (14)	C22—C21—C27	118.6 (4)
O6—Zn1—O1	90.79 (12)	C22—C21—C20	122.0 (4)
O3—Zn1—O1	90.58 (13)	C27—C21—C20	119.4 (4)
O6—Zn1—O5	87.57 (13)	C21—C22—N2	118.0 (4)
O3—Zn1—O5	85.48 (13)	C21—C22—C24	120.5 (4)
O1—Zn1—O5	171.47 (15)	N2—C22—C24	121.4 (4)
O6—Zn1—O7	115.15 (14)	N2—C23—C19	125.1 (4)
O3—Zn1—O7	103.44 (13)	N2—C23—H23A	117.5
O1—Zn1—O7	95.14 (15)	C19—C23—H23A	117.5
O5—Zn1—O7	93.13 (15)	C25—C24—C22	121.4 (4)
O2—C1—O1	123.3 (4)	C25—C24—H24A	119.3
O2—C1—C2	117.9 (4)	C22—C24—H24A	119.3
O1—C1—C2	118.8 (4)	C24—C25—N4	123.9 (4)
C6—C2—C3	118.0 (4)	C24—C25—C26	116.1 (4)
C6—C2—C1	116.6 (4)	N4—C25—C26	119.9 (4)
C3—C2—C1	125.4 (4)	C27—C26—F2	117.8 (4)
O3—C3—C2	125.3 (4)	C27—C26—C25	124.1 (4)
O3—C3—C4	118.7 (4)	F2—C26—C25	118.1 (3)
C2—C3—C4	116.0 (4)	C26—C27—C21	119.2 (4)
C12—C4—C5	118.3 (4)	C26—C27—H27A	120.4
C12—C4—C3	119.9 (4)	C21—C27—H27A	120.4
C5—C4—C3	121.8 (4)	N4—C28—C29	108.7 (4)
N1—C5—C4	118.9 (4)	N4—C28—H28A	110.0
N1—C5—C9	120.8 (4)	C29—C28—H28A	110.0
C4—C5—C9	120.2 (4)	N4—C28—H28B	110.0
N1—C6—C2	125.7 (4)	C29—C28—H28B	110.0
N1—C6—H6A	117.1	H28A—C28—H28B	108.3
C2—C6—H6A	117.1	N5—C29—C28	111.8 (4)
N1—C7—C8	112.8 (4)	N5—C29—H29A	109.3
N1—C7—H7A	109.0	C28—C29—H29A	109.3
C8—C7—H7A	109.0	N5—C29—H29B	109.3
N1—C7—H7B	109.0	C28—C29—H29B	109.3
C8—C7—H7B	109.0	H29A—C29—H29B	107.9
H7A—C7—H7B	107.8	N5—C30—C31	110.1 (4)
C7—C8—H8A	109.5	N5—C30—H30A	109.6
C7—C8—H8B	109.5	C31—C30—H30A	109.6
H8A—C8—H8B	109.5	N5—C30—H30B	109.6
C7—C8—H8C	109.5	C31—C30—H30B	109.6
H8A—C8—H8C	109.5	H30A—C30—H30B	108.2
H8B—C8—H8C	109.5	N4—C31—C30	111.0 (4)
C10—C9—C5	120.9 (4)	N4—C31—H31A	109.4
C10—C9—H9A	119.6	C30—C31—H31A	109.4
C5—C9—H9A	119.6	N4—C31—H31B	109.4
C9—C10—N3	123.0 (4)	C30—C31—H31B	109.4
C9—C10—C11	116.9 (4)	H31A—C31—H31B	108.0
N3—C10—C11	120.1 (4)	N5—C32—H32A	109.5
C12—C11—F1	119.1 (4)	N5—C32—H32B	109.5
C12—C11—C10	122.9 (4)	H32A—C32—H32B	109.5
F1—C11—C10	118.0 (4)	N5—C32—H32C	109.5
C11—C12—C4	120.8 (4)	H32A—C32—H32C	109.5
C11—C12—H12A	119.6	H32B—C32—H32C	109.5
C4—C12—H12A	119.6	N2—C33—C34	112.1 (4)
N3—C13—C14	109.9 (4)	N2—C33—H33A	109.2
N3—C13—H13A	109.7	C34—C33—H33A	109.2
C14—C13—H13A	109.7	N2—C33—H33B	109.2
N3—C13—H13B	109.7	C34—C33—H33B	109.2
C14—C13—H13B	109.7	H33A—C33—H33B	107.9
H13A—C13—H13B	108.2	C33—C34—H34A	109.5
N6—C14—C13	111.0 (4)	C33—C34—H34B	109.5
N6—C14—H14A	109.4	H34A—C34—H34B	109.5
C13—C14—H14A	109.4	C33—C34—H34C	109.5
N6—C14—H14B	109.4	H34A—C34—H34C	109.5
C13—C14—H14B	109.4	H34B—C34—H34C	109.5
H14A—C14—H14B	108.0	C6—N1—C5	119.4 (4)
N3—C15—C16	112.4 (4)	C6—N1—C7	118.8 (3)
N3—C15—H15A	109.1	C5—N1—C7	121.8 (4)
C16—C15—H15A	109.1	C23—N2—C22	119.9 (4)
N3—C15—H15B	109.1	C23—N2—C33	119.2 (3)
C16—C15—H15B	109.1	C22—N2—C33	120.9 (3)
H15A—C15—H15B	107.9	C10—N3—C13	116.8 (4)
N6—C16—C15	111.5 (4)	C10—N3—C15	115.3 (4)
N6—C16—H16A	109.3	C13—N3—C15	112.3 (4)
C15—C16—H16A	109.3	C25—N4—C28	118.1 (3)
N6—C16—H16B	109.3	C25—N4—C31	116.8 (3)
C15—C16—H16B	109.3	C28—N4—C31	110.2 (4)
H16A—C16—H16B	108.0	C30—N5—C29	108.8 (4)
N6—C17—H17A	109.5	C30—N5—C32	109.9 (4)
N6—C17—H17B	109.5	C29—N5—C32	110.2 (5)
H17A—C17—H17B	109.5	C14—N6—C17	112.5 (5)
N6—C17—H17C	109.5	C14—N6—C16	109.2 (4)
H17A—C17—H17C	109.5	C17—N6—C16	112.4 (5)
H17B—C17—H17C	109.5	C1—O1—Zn1	130.2 (3)
O4—C18—O5	122.9 (4)	C3—O3—Zn1	124.2 (3)
O4—C18—C19	119.5 (4)	C18—O5—Zn1	133.8 (3)
O5—C18—C19	117.6 (4)	C20—O6—Zn1	128.6 (3)
C23—C19—C20	118.7 (4)	Zn1—O7—H7	105 (4)
C23—C19—C18	117.1 (4)	Zn1—O7—H8	103 (5)
C20—C19—C18	124.2 (4)	H7—O7—H8	105 (2)
O6—C20—C19	127.0 (4)	H8WA—O8—H8WB	104.6
O6—C20—C21	117.0 (4)	H9WA—O9—H9WB	143.6
C19—C20—C21	116.0 (4)		
			
O2—C1—C2—C6	8.5 (6)	N5—C30—C31—N4	58.4 (5)
O1—C1—C2—C6	−170.6 (4)	C2—C6—N1—C5	1.0 (7)
O2—C1—C2—C3	−173.3 (5)	C2—C6—N1—C7	−179.3 (4)
O1—C1—C2—C3	7.6 (7)	C4—C5—N1—C6	0.2 (6)
C6—C2—C3—O3	−177.9 (4)	C9—C5—N1—C6	−178.2 (4)
C1—C2—C3—O3	3.9 (7)	C4—C5—N1—C7	−179.5 (4)
C6—C2—C3—C4	2.9 (6)	C9—C5—N1—C7	2.0 (6)
C1—C2—C3—C4	−175.3 (4)	C8—C7—N1—C6	−101.6 (5)
O3—C3—C4—C12	−2.7 (6)	C8—C7—N1—C5	78.1 (5)
C2—C3—C4—C12	176.6 (4)	C19—C23—N2—C22	1.1 (7)
O3—C3—C4—C5	178.8 (4)	C19—C23—N2—C33	−179.8 (4)
C2—C3—C4—C5	−1.9 (6)	C21—C22—N2—C23	−3.3 (6)
C12—C4—C5—N1	−178.2 (4)	C24—C22—N2—C23	178.3 (4)
C3—C4—C5—N1	0.3 (6)	C21—C22—N2—C33	177.5 (4)
C12—C4—C5—C9	0.3 (6)	C24—C22—N2—C33	−0.9 (6)
C3—C4—C5—C9	178.8 (4)	C34—C33—N2—C23	−95.9 (5)
C3—C2—C6—N1	−2.7 (7)	C34—C33—N2—C22	83.2 (5)
C1—C2—C6—N1	175.7 (4)	C9—C10—N3—C13	−16.3 (7)
N1—C5—C9—C10	179.2 (4)	C11—C10—N3—C13	162.5 (4)
C4—C5—C9—C10	0.7 (7)	C9—C10—N3—C15	119.0 (5)
C5—C9—C10—N3	178.0 (4)	C11—C10—N3—C15	−62.3 (6)
C5—C9—C10—C11	−0.8 (7)	C14—C13—N3—C10	−170.1 (4)
C9—C10—C11—C12	−0.2 (7)	C14—C13—N3—C15	53.3 (6)
N3—C10—C11—C12	−179.1 (4)	C16—C15—N3—C10	171.2 (4)
C9—C10—C11—F1	178.6 (4)	C16—C15—N3—C13	−51.6 (6)
N3—C10—C11—F1	−0.3 (7)	C24—C25—N4—C28	−11.3 (6)
F1—C11—C12—C4	−177.5 (4)	C26—C25—N4—C28	164.6 (4)
C10—C11—C12—C4	1.3 (7)	C24—C25—N4—C31	123.7 (5)
C5—C4—C12—C11	−1.3 (7)	C26—C25—N4—C31	−60.4 (5)
C3—C4—C12—C11	−179.8 (4)	C29—C28—N4—C25	−165.5 (4)
N3—C13—C14—N6	−58.7 (6)	C29—C28—N4—C31	56.7 (5)
N3—C15—C16—N6	53.0 (7)	C30—C31—N4—C25	163.9 (4)
O4—C18—C19—C23	3.3 (6)	C30—C31—N4—C28	−57.8 (5)
O5—C18—C19—C23	−177.6 (4)	C31—C30—N5—C29	−58.7 (5)
O4—C18—C19—C20	−174.7 (4)	C31—C30—N5—C32	−179.4 (5)
O5—C18—C19—C20	4.3 (7)	C28—C29—N5—C30	60.1 (6)
C23—C19—C20—O6	175.3 (4)	C28—C29—N5—C32	−179.4 (5)
C18—C19—C20—O6	−6.6 (7)	C13—C14—N6—C17	−174.2 (5)
C23—C19—C20—C21	−6.0 (6)	C13—C14—N6—C16	60.2 (6)
C18—C19—C20—C21	172.1 (4)	C15—C16—N6—C14	−57.2 (6)
O6—C20—C21—C22	−177.3 (4)	C15—C16—N6—C17	177.2 (6)
C19—C20—C21—C22	3.8 (6)	O2—C1—O1—Zn1	−175.5 (4)
O6—C20—C21—C27	3.9 (6)	C2—C1—O1—Zn1	3.5 (6)
C19—C20—C21—C27	−174.9 (4)	O6—Zn1—O1—C1	−157.3 (4)
C27—C21—C22—N2	179.5 (4)	O3—Zn1—O1—C1	−16.2 (4)
C20—C21—C22—N2	0.8 (6)	O7—Zn1—O1—C1	87.4 (4)
C27—C21—C22—C24	−2.1 (6)	C2—C3—O3—Zn1	−24.5 (6)
C20—C21—C22—C24	179.2 (4)	C4—C3—O3—Zn1	154.6 (3)
C20—C19—C23—N2	3.8 (7)	O6—Zn1—O3—C3	118.0 (3)
C18—C19—C23—N2	−174.4 (4)	O1—Zn1—O3—C3	26.0 (3)
C21—C22—C24—C25	1.7 (6)	O5—Zn1—O3—C3	−161.5 (3)
N2—C22—C24—C25	−179.9 (4)	O7—Zn1—O3—C3	−69.4 (3)
C22—C24—C25—N4	176.9 (4)	O4—C18—O5—Zn1	−173.9 (3)
C22—C24—C25—C26	0.9 (6)	C19—C18—O5—Zn1	7.1 (7)
C24—C25—C26—C27	−3.4 (7)	O6—Zn1—O5—C18	−11.8 (5)
N4—C25—C26—C27	−179.6 (4)	O3—Zn1—O5—C18	−153.5 (5)
C24—C25—C26—F2	176.2 (4)	O7—Zn1—O5—C18	103.3 (5)
N4—C25—C26—F2	0.0 (6)	C19—C20—O6—Zn1	−2.7 (7)
F2—C26—C27—C21	−176.5 (4)	C21—C20—O6—Zn1	178.6 (3)
C25—C26—C27—C21	3.1 (7)	O3—Zn1—O6—C20	88.7 (4)
C22—C21—C27—C26	−0.3 (6)	O1—Zn1—O6—C20	−179.4 (4)
C20—C21—C27—C26	178.5 (4)	O5—Zn1—O6—C20	9.0 (4)
N4—C28—C29—N5	−59.3 (6)	O7—Zn1—O6—C20	−83.4 (4)

### Hydrogen-bond geometry (Å, °)

**Table:**

*D*—H···*A*	*D*—H	H···*A*	*D*···*A*	*D*—H···*A*
O7—H7···O5^i^	0.85 (1)	1.79 (1)	2.638 (5)	176 (6)
O8—H8WA···N5	0.85	2.21	3.063 (9)	179
O8—H8WB···O1^ii^	0.85	2.20	3.054 (7)	179
O9—H9WA···N6	0.85	1.99	2.843 (9)	180

Symmetry codes: (i) −*x*+1, −*y*−1, −*z*+1; (ii) −*x*+2, −*y*−1, −*z*+2.
